# Novel AIP mutation in exon 6 causing acromegaly in a German family

**DOI:** 10.1007/s40618-023-02031-5

**Published:** 2023-02-09

**Authors:** M. Detomas, B. Altieri, J. Flitsch, W. Saeger, M. Korbonits, T. Deutschbein

**Affiliations:** 1 Department of Internal Medicine I, Division of Endocrinology and Diabetes, University Hospital Würzburg, University of Würzburg, Würzburg, Germany; 2 Department of Neurosurgery, University Medical Center Hamburg-Eppendorf, Hamburg, Germany; 3 Institute of Neuropathology, University of Hamburg, UKE, Hamburg, Germany; 4 Centre for Endocrinology, Barts and the London School of Medicine and Dentistry, William Harvey Research Institute, Queen Mary University of London, London, UK; 5 Medicover Oldenburg MVZ, Oldenburg, Germany

**Keywords:** AIP, Acromegaly, Colorectal cancer, Familial, Gene, GH, Growth hormone, Mutation, Pituitary adenoma, Twins

## Abstract

The most frequent genetic alteration of familial isolated growth hormone producing pituitary neuroendocrine tumors is a germline mutation of the aryl hydrocarbon receptor-interacting protein (AIP*)* gene. Various *AIP* mutations are already known; however, an *AIP* mutation in exon 6 (c.811_812del; p.Arg271Glyfs*16) has not been reported yet. Here, we report a German family with two identical twins who were both affected by acromegaly and carried the above-mentioned novel *AIP* mutation. The father was found to be an unaffected carrier, while the paternal aunt most likely suffered from acromegaly as well and died from metastatic colorectal cancer. Apart from reporting a novel *AIP* mutation, this study does not only highlight the different clinical and histological features of the *AIP* mutated growth hormone producing pituitary neuroendocrine tumors but also confirms the poor responsiveness of dopamine agonists in *AIP* mutated acromegaly. Furthermore, it highlights the increased mortality risk of comorbidities typically associated with acromegaly.

## Rapid communication

Acromegaly is a rare disorder usually due to growth hormone (GH) excess caused by a pituitary adenoma. The most frequent genetic alteration, being responsible for about 22% of familial isolated GH-producing pituitary adenoma and around 4% of sporadic acromegaly, is a germline mutation of the aryl hydrocarbon receptor-interacting protein (*AIP)* gene [1, 2]. A heterozygous germline *AIP* mutation (c.646G > T; p.E216*) has previously been reported in non-identical female twins, each with a silent somatotroph pituitary macroadenoma [3]. Identical twins with a de novo* AIP* mutation (c.910C < T; p.R304*) have also been reported, with one twin suffering from gigantism, while the other being an unaffected carrier [4]. Furthermore, acromegaly occurring in one of two uniovular twins (who did not undergo genetic workup) [5, 6] or in male twins (to our knowledge both affected by an *AIP* mutation) have sporadically been reported [7, 8]. Here, we report the case of identical male twins with active acromegaly and a novel *AIP* mutation in exon 6 (c.811_812del; p.Arg271Glyfs*16).

**Fig. 1:**
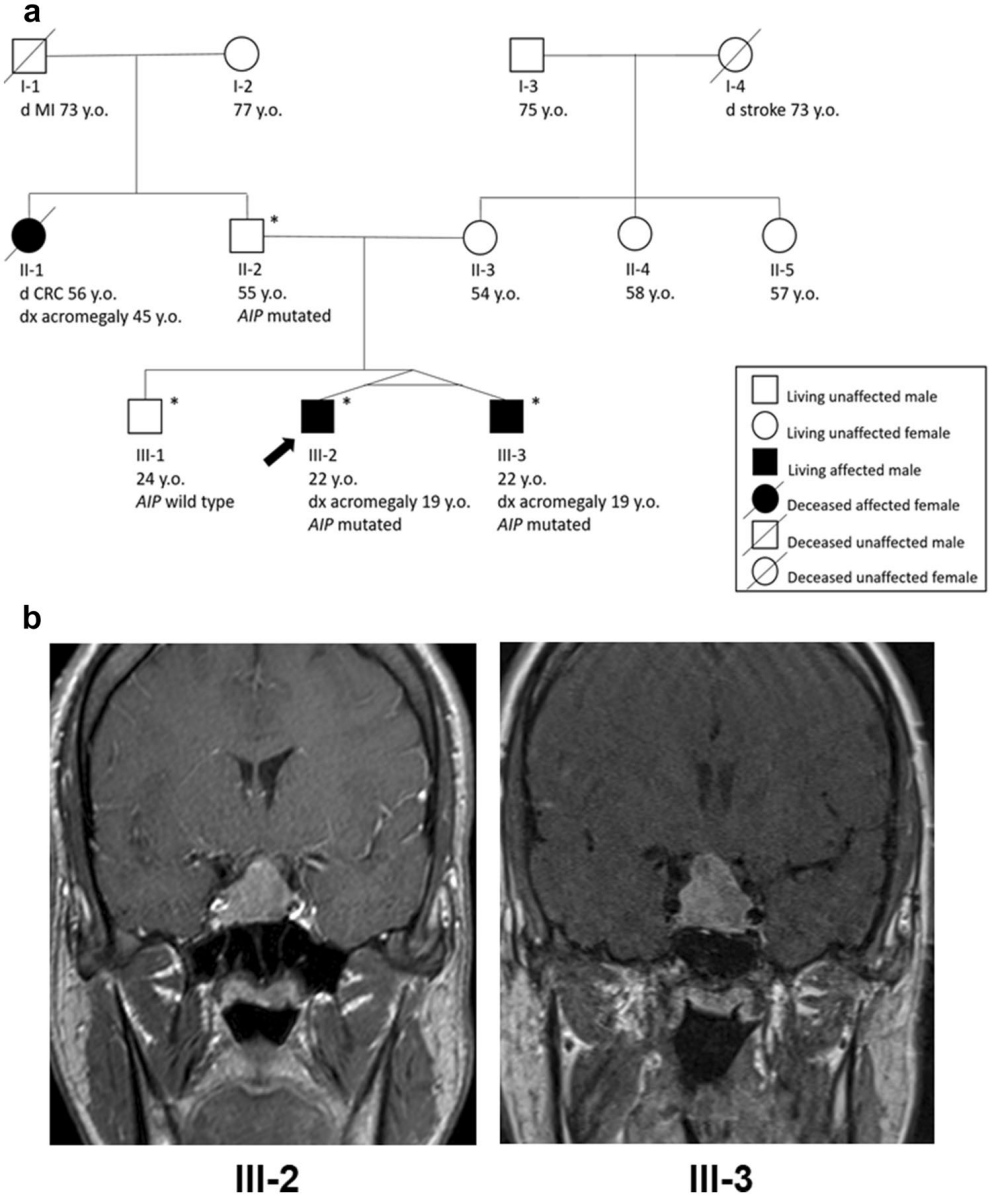
**a** Pedigree of the family. The arrow indicates the proband (subject III-2), while the asterisk indicate the patients undergoing genetic workup for *AIP* mutational status. The identified *AIP* mutation is c.811_812del; p.Arg271Glyfs*16. The provided ages are those at death, at the initial diagnosis of acromegaly, or at last follow-up. **b** Coronal T1-weighted magnetic resonance image with contrast medium of the sellar region of patient III-2 (left) and patient III-3 (right) *CRC* colorectal cancer, *d* age at patient´s death, *dx* age at diagnosis of acromegaly, *MI* myocardial infarction, *y.o.* years old

In April 2019, a 19-year-old male patient (patient III-2 in Fig. 1a) underwent a brain magnetic resonance imaging (MRI) because of recurrent headaches. Radiological workup revealed a pituitary macroadenoma of 2.5 × 2.1 × 2.0 cm (Fig. 1b). At initial presentation at our endocrine outpatient clinic, the patient was 200 cm tall and had a body weight of 114 kg. Apart from headaches, no complaints were reported. The patient was not on medication at that time. While the patient’s own medical history only contained a wrist fracture during childhood, the paternal aunt was treated with a dopamine agonist (bromocriptine) because of a suspected prolactinoma. She was at that time 54 years old and also under chemotherapy because of metastatic colorectal cancer.

During physical examination, our index patient presented with facial and acral dysmorphia. Hormonal analysis revealed an insulin-like growth factor 1 (IGF-1) of 728 µg/l (1.8 × upper limit of normal (ULN)), a random GH of 16.0 µg/l (normal range 0–5), and a prolactin of 26.9 µg/l (normal range 7.1–15.7). A subsequent oral glucose tolerance test confirmed acromegaly (insufficient GH decrease from 15.8 µg/l to 13.40 µg/l after 2 h, normal range ≤ 0.4 µg/l). Of note, the other pituitary hormones were normal (Table 1).

**Table 1:** Clinical, hormonal, and genetic characteristics of the investigated family members at the time of their initial diagnostic workup at our endocrine outpatient clinic

	III-2 (Proband)	III-3 (Twin brother)	II-2 (Father)	II-1 (Paternal aunt)
Clinical characteristics				
Age	19	19	53	45
Size (cm)	200	201	194	184
Weight (kg)	114	100	100	74
BMI (kg/m^2^)	28.5	24.8	26.7	21.9
Hormonal parameters				
IGF-1 (µg/l)	**728**	**554**	201	**365**
Random GH (µg/l)	**16.0**	**17.4**	0.7	**6.5**
Prolactin (µg/l)	**26.9**	**30.8**	12.6	**26.4**
TSH (mlU/l)	1.3	1.4	1.2	1.6
FT4 (pmol/l)	12.5	**10.3**	13.8	17.1
ACTH (ng/l)	45.1	27.5	14.9	22.4
Cortisol (µg/dl)	12.2	**3.9**	18.6	17.3
LH (IU/l)	3.0	5.2	6.7	6.6
FSH (IU/l)	5.3	3.8	10.0	5.4
Testosterone (µg/dl)	2.0	3.5	3.9	0.4
Estradiol (ng/l)	n.a.	n.a.	n.a.	42
Genetic analysis				
AIP gene status	Mutated	Mutated	Mutated	n.a.

Values reported in bold are pathological

*ACTH* adrenocorticotropin, *AIP* aryl hydrocarbon receptor-interacting protein gene, *BMI* body mass index, *FSH* follicle-stimulating hormone, *fT4* free thyroxine, *GH* growth hormone, *IGF-1* insulin-like growth factor 1, *LH* luteinizing hormone, *n.a.* not available, *TSH* thyroid stimulating hormone

**Fig. 2:**
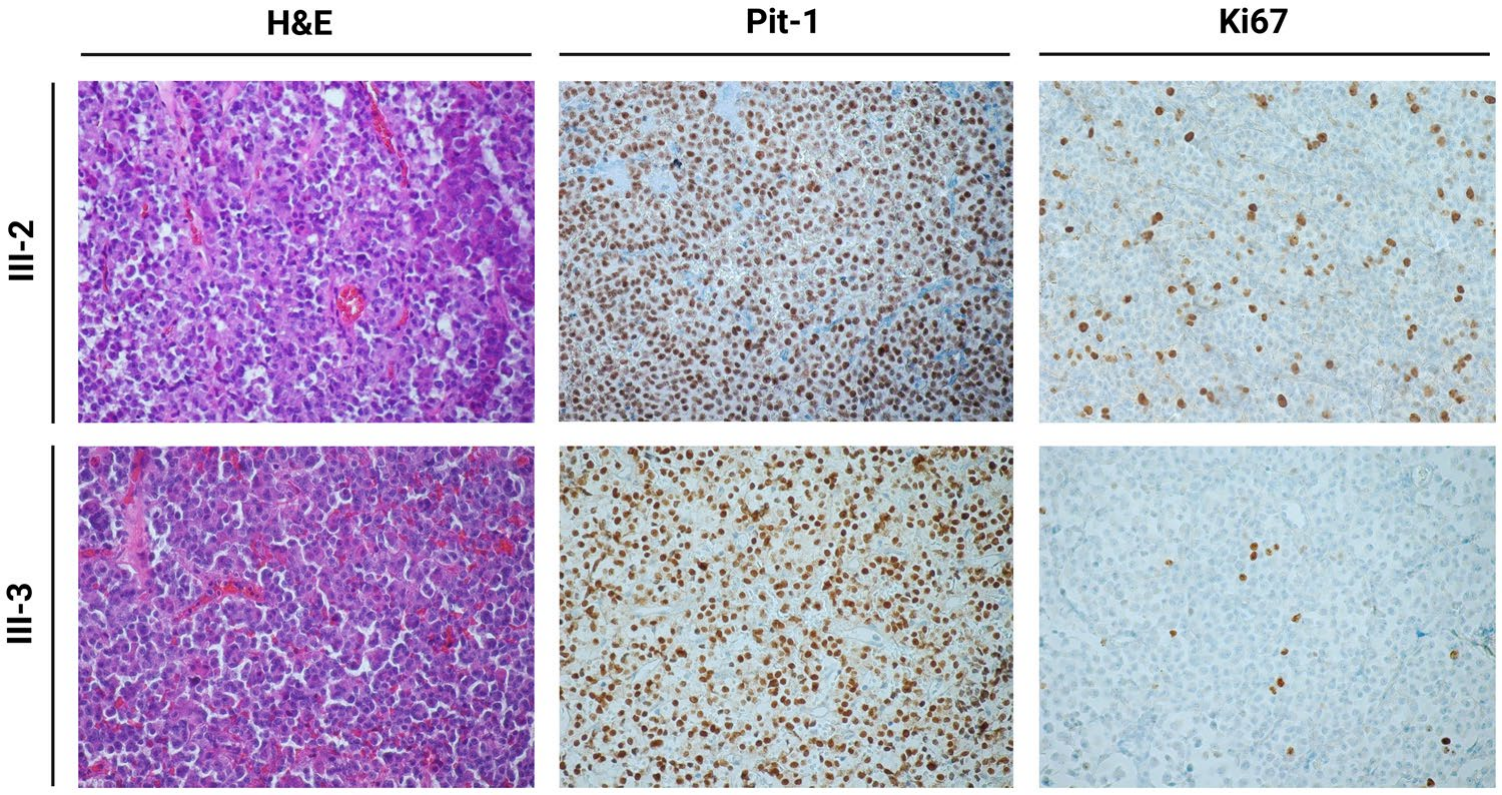
Staining for hematoxylin and eosin, Pit-1, and Ki67 in both twins’ growth hormone-secreting pituitary neuroendocrine tumors. The main difference was observed for Ki67 (up to 10% in patient III-2, facing 5% in patient III-3). *H&E* hematoxylin and eosin

One month later, the patient was operated in a tertiary neurosurgical referral center for sellar masses. The neuropathological examination confirmed presence of a somatotroph pituitary neuroendocrine tumor (along with strong hormonal expression, sparse granulation, globular expression of Cam.5.2, fibrous bodies in more than 70% of tumor cells, Ki67 ranging from 5 to 10% in some hotspots, and strongly expressed p53 in 5% of the cells; Pit-1 was positive, whereas T-pit and SF-1 were negative) (Fig. 2). According to the WHO classification from 2021, this tumor was described as an aggressive pituitary adenoma [9]. If a clinicopathological classification originally suggested by Trouillas et al. was used, the tumor was categorized as invasive and proliferative (grade 2b) [10].

Because of the invasion of the right cavernous sinus (see Fig. 1b), a residual tumor persisted after surgery, and the patient remained biochemically uncontrolled (IGF-1: 424 µg/l, 1.3 × ULN; random GH: 2.6 µg/l). Accordingly, a first-generation long-acting somatostatin analog was introduced (lanreotide 120 mg as i.m. injection every 4 weeks) but caused persistent nausea and fatigue. In January 2021, the patient therefore underwent a cyberknife stereotactic radiosurgery of the residual tumor. By June 2022, both IGF-1 (to 242 µg/l) and GH (to 0.7 µg/l) normalized without drug therapy. In September 2022, an asymptomatic minimal increase of IGF-1 to 336 µg/l (1.1 × ULN) was observed. Accordingly, a dopamine agonist (cabergoline) was initiated, and the dose was progressively increased from 0.25 mg to 0.5 mg twice weekly. After 6 weeks of therapy, however, the IGF-1 further increased to 409 µg/l (1.3 × ULN), resulting in a therapeutic switch from cabergoline to a GH receptor antagonist (pegvisomant, introduced with an initial 80 mg s.c. injection which was followed by daily s.c. injections of each 10 mg).

Because of the patient’s young age at initial presentation of the somatotroph pituitary tumor, a germline genetic analysis was performed. A novel *AIP* mutation in exon 6 (c.811_812del; p.Arg271Glyfs*16) was identified. As a consequence, first-degree family members were screened for this mutation as well. The identical twin brother and the father were both found to be carriers of the same mutation, while the twins’ older brother was having a wild-type *AIP* gene status.

The patient’s twin brother (patient III-3 in Fig. 1a) was referred to our endocrine outpatient clinic for the first time in June 2019. He did not have any complaints, was not on medical treatment, and had an inconspicuous medical history. The patient was 201 cm tall and weighed 100 kg. Physical examination revealed modest facial dysmorphia. However, no other signs and symptoms of acromegaly were detectable. IGF-1 (554 µg/d, 1.3 × ULN), random GH (17.40 µg/l), and prolactin (30.8 µg/l) were all elevated, whereas fT4 and basal serum cortisol were slightly decreased. All other pituitary hormones were normal (Table 1). Secondary adrenal insufficiency was confirmed with a pathological ACTH stimulation test (serum cortisol at 60 min 12.0 µg/dl, normal range ≥ 20.0 µg/dl). Accordingly, hydrocortisone and l-thyroxin replacement therapy were initiated. A 2.4 × 2.3 × 1.7 cm pituitary macroadenoma was identified in a sellar MRI (Fig. 1b). Following transsphenoidal surgery, the hormonal profile normalized (IGF 256 µg/l; random GH 0.8 µg/l; prolactin 3.9 µg/l). The neuropathologist described a pituitary neuroendocrine tumor with low-to-medium expression of somatotroph cells and negative staining for prolactin, ACTH, TSH, and LH. Further neuropathological workup of the tumor revealed sparse granulation, globular expression of Cam.5.2 with fibrous bodies in more than 70% of tumor cells, Ki67 5%, strong expression of p53 in 5% of the cells; Pit-1 was positive, whereas T-pit and SF-1 were negative (Fig. 2). These data were also consistent with an aggressive pituitary adenoma [9], or an invasive and proliferative tumor (grade 2b) [10].

In June 2020, we investigated the twins’ father (subject II-2 in Fig. 1a), who was 194 cm tall (mid-parental height: 188 cm). The father (at that time 53 years old) had refused any endocrine workup before, since he did not have any complaints and biochemical workup (including IGF-1 and random GH) performed by the general practitioner had been normal. We also measured pituitary hormones in the normal range (e.g., IGF-1: 201 µg/dl; random GH: 0.7 µg/l; prolactin: 12.6 µg/l; testosterone: 3.9 µg/l, normal range 1.6–9.0) (Table 1). Because of his two affected sons and the results of the genetic workup, a pituitary MRI was performed (without any pituitary abnormalities). The paternal grandmother refused genetic testing.

The twins’ paternal aunt (patient II-1 in Fig. 1a.) unfortunately died from metastatic colorectal cancer in 2021, at the age of 56. Interestingly, we noticed that she was referred to our endocrine outpatient clinic because of facial and acral dysmorphia already in March 2010. At that time, elevated levels of IGF-1 (365 µg/l, 1.4 × ULN) and random GH (6.47 µg/l) were observed, along with a slightly elevated prolactin of 26.4 µg/l and a body size of 184 cm. Of note, however, the biochemical evaluation was performed under 7.5 mg bromocriptine per day. This treatment had already been initiated elsewhere because of hyperprolactinemia. Unfortunately, the patient never came back to our institution despite further diagnostic measures were imperatively suggested.

In conclusion, we here report a novel *AIP* mutation in exon 6, which was responsible for acromegaly in two homozygous twins, and possibly in their paternal aunt. A mutation affecting the same nucleotide at c.811 (missense change c.811C > T; p.Arg271Trp) was already reported repeatedly in the context of familial isolated pituitary adenomas with GH-hypersecretion [11, 12], and indeed, it represents one of the genetic hotspots in this gene. This locus of the *AIP* gene can therefore be considered as important in the pathophysiological context of acromegaly.

The impact of the novel *AIP* mutation in exon 6 (c.811_812del; p.Arg271Glyfs*16) on the phenotype remains to be elucidated, since it caused a variable spectrum of clinical impairment and tumor-aggressiveness in the affected family members. Recently, it has been hypothesized that methylation processes may have a relevant influence on the variable behavior of pituitary neuroendocrine tumors [13], and this may have been true also for our case III-2. Considering that methylation of the *GSTP1* gene may be associated with an increased resistance to somatostatin analogues [14], resistance to dopamine agonists (as observed here and in other studies [15, 16]) may be explained as well. Finally, this report highlights the importance of early diagnosis and adequate treatment of GH excess, since some comorbidities related to acromegaly (e.g., colorectal cancer) may be lethal.

## Data Availability

The authors declare that the data supporting the findings of this study are available within the article.
